# Mothers’ accounts of the impact of being in nature on postnatal wellbeing: a focus group study

**DOI:** 10.1186/s12905-023-02165-x

**Published:** 2023-01-23

**Authors:** Katherine Hall, Jonathan Evans, Rosa Roberts, Richard Brown, Christopher Barnes, Katrina Turner

**Affiliations:** 1 Centre for Academic Mental Health, Population Health Sciences, Bristol Medical School, University of Bristol, Bristol, UK; 2 Avon and Wiltshire Partnership NHS Trust, Bath, UK; 3 Oxford Health NHS Foundation Trust, Oxford, UK; 4 School of Psychology, College of Health, Psychology and Social Care, University of Derby, Derby, UK; 5 Centre for Academic Mental Health and Centre for Academic Primary Care, Population Health Sciences, Bristol Medical School, University of Bristol, Bristol, UK

**Keywords:** Postnatal depression, Nature, Outdoors, Nature-based interventions, Mothers

## Abstract

**Background:**

The postnatal period is a vulnerable time for mothers to experience stress and mental health difficulties. There is increasing evidence that spending time in nature is beneficial for wellbeing. Nature-based interventions have been developed to support mental health, but not specifically tailored for mothers during the postnatal period. Understanding mothers’ views and experiences of nature would help determine the suitability for and potential impact of such interventions on postnatal wellbeing.

**Aims:**

To explore mothers’ views on the impact of spending time in nature on their postnatal mental wellbeing.

**Methods:**

Focus groups were held with mothers of young children (under five), including mothers from migrant and refugee communities, mothers living with mental health difficulties, and disabled mothers. Data were analysed using reflexive thematic analysis.

**Results:**

Four focus groups were held, with a total of 30 participants. Six themes were developed: (1) mothers’ experiences of what constitutes ‘nature’; (2) sensing nature improves wellbeing; (3) natural spaces facilitate human connection; (4) nature provides escape and relief from daily indoor stressors; (5) nature allows new perspectives; and (6) mothers face a variety of environmental, practical, psychological, physical, socioeconomic, and cultural barriers to spending time in nature during the postnatal period.

**Conclusions:**

Mothers report significant benefits to their postnatal wellbeing when spending time in nature. Further research is warranted to understand whether nature-based interventions have the potential to support postnatal wellbeing, socially, mentally, and physically.

## Background

Modern living and urbanisation have been associated with pressing environmental and public health challenges [1–3]. These include rising rates of mental health difficulties, including those occurring in the postnatal period (variously defined as either the first 12- or 24-months following childbirth). High prevalence rates of postnatal depression and anxiety have been reported (around 15–20% [4] and 10% [5, 6] respectively), with detrimental consequences to mothers, families, infants and wider society [7]. Furthermore, these issues frequently go undiagnosed, especially in under-represented groups such as mothers from ethnic minority backgrounds or with disabilities [8, 9].

Existing evidence and guidelines support the use of psychosocial, psychological, and pharmacological interventions for common postnatal mental health difficulties [10, 11]. However, many women worry about taking medication during the postnatal period, due to possible side effects or infant exposure via breastmilk [12]. As such, non-pharmacological treatment may be more acceptable. Unfortunately, long waiting lists and childcare commitments render psychological therapies inaccessible to many mothers in need. Numerous barriers to receiving accessible and available healthcare mean that very few mothers receive treatment, despite presenting with clinical symptoms [14]. Moreover, people from racial and ethnic minority groups are often less likely to receive treatment compared to White British groups, a problem not specific to the postnatal period [13].

One of the ways in which women and families may choose to care for their own wellbeing is through spending time in nature. The important role of the natural environment in maintaining and improving mental and physical health has been well-established (in non-perinatal populations) across age ranges, cultures, and social class [15–18]. Amongst other benefits, studies have found that time spent in nature improves mood and emotional regulation [19–21], reduces symptoms of stress anxiety [22], improves sleep [23–26], encourages optimal child development [27], and enhances creativity [28, 29], resilience [30] and social cohesion [31, 32]. Indeed, a close relationship with nature is considered by some a basic psychological need and a pre-requisite to wellbeing [18, 33, 34].

Contact with nature has the potential to reduce health inequalities, given evidence of particular advantages to low-income populations [35–38]. It is also possible that nature-based interventions may have higher cross-cultural validity than current treatment modalities (for example Cognitive Behavioural Therapy), and improve access to care for racial and ethnic minority groups. There is some indication that nature may act as a coping mechanism for refugees who are integrating into the UK [39], or for people worldwide to manage distress during the coronavirus pandemic [40], including mothers of young children [41]. Nature-based interventions may potentially represent a sustainable option in comparison to traditional treatments, and may be more cost-effective [42], both important factors to consider when developing health interventions in our current economic and ecological climate.

However, there is limited understanding about the role of nature in supporting postnatal mental health. The aim of this study was to explore mothers’ views about the effect of spending time in nature during the postnatal period, its impact on their wellbeing, and the challenges they face when trying to access natural environments.

## Methods

### Study design

This exploratory focus group study was based in Bristol, UK. Focus groups were held with mothers of young children who self-reported experiencing postnatal mental health difficulties. They were also held with refugee mothers, migrant mothers and disabled mothers, whether or not they reported postnatal mental health difficulties. This method of data collection was chosen as an accessible, flexible method of facilitating interactive discussion and understanding shared cultural discourses [43, 44]. It was informed by a critical realist and inductive approach, with data conceptualised as reflecting, at least to some degree, the mothers’ relationships with and experiences within the natural world, which are understood to be shaped through culturally and socially available meanings and constructions related to ‘nature’, motherhood and wellbeing [45]. The groups were conducted as part of a larger study that aimed to develop a nature-based programme to support postnatal wellbeing.

### Recruitment and sampling

To elicit a diversity of opinion, we set out to recruit mothers from the following groups:

1. mothers self-reporting common postnatal mental health difficulties
2. refugee mothers
3. migrant mothers
4. disabled mothers

Eligible mothers needed to be over 18 years of age and have an infant or young child (under the age of 5). Participants were required to speak English, except those in the focus group for refugee women, for which an interpreter was available.

An advert detailing the aims of the study and how those interested could take part was developed, with four versions each tailored to the specific groups described above. The respective advert was disseminated through the social media channels of a perinatal mental health charity, to a refugee centre, to an ethnically diverse mother-and-baby group, and through social media channels for disabled mothers. Women contacting the research team were given further information about the study and asked to sign and return a consent form, if they wanted to take part, as well as a brief demographic questionnaire. This included a question about whether the participant self-reported experiencing any postnatal mental health difficulties. Mothers for focus group 1 were recruited from a charity supporting women with postnatal mental health difficulties, which screened all of their service users for depression and anxiety using the Edinburgh Postnatal Depression Scale (EPDS) and Generalised Anxiety Disorder Assessment (GAD-7). We did not conduct our own screening, as our objective was not to categorise mothers’ levels of distress. Rather, we aimed to understand whether contact with nature may represent a relevant strategy in the treatment or selective prevention of postnatal mental health difficulties (i.e., targeting specific subgroups of mothers that are believed to be at higher risk).

### Data collection

A flexible topic guide was developed to achieve consistency across the focus groups. It was based on the aims of the group, relevant literature, team discussions and input from wider stakeholders, for example mothers with lived experience of postnatal mental health difficulties and charity providers working with mothers from a diverse range of backgrounds. It included questions aiming to elicit information on whether and how the participants spent time in nature, what effect they thought nature had on their and their baby’s wellbeing (if any), and what challenges they faced in accessing nature.

Focus groups were facilitated by KH, a psychiatry doctor who received prior training in qualitative methods, alongside another member of the research team. KH had secured ethical approval to conduct groups in person or online, depending on participants’ situations and COVID-19 guidelines at the time. The focus group for refugee women was conducted mostly in Arabic (with some participants speaking English), in the presence of an interpreter. For all groups, babies were allowed to be present. The groups were audio-recorded with participant consent.

### Data analysis

Audio-recordings were transcribed verbatim by University of Bristol-approved professional transcribers, and checked for accuracy by KH and RB. For the focus group conducted in Arabic, the original spoken Arabic was transcribed directly into English prior to analysis, by the same Transcription and Translation Service.

Reflexive thematic analysis was chosen to identify patterns of meaning across the data [46, 47]. Our approach was inductive and data-driven [48]. The six phases of Braun and Clarke’s reflective thematic analysis were used through an iterative process [46, 49]. This involved (1) KH listening and re-listening to all recordings, and KH, RR and RB reading and re-reading all transcripts, noting reflections, (2) KH, RR and RB coding data line-by-line, (3) codes being clustered, mind-mapped (by KH) and tabulated (by RR and RB), with regular team discussion, (4) developing and reviewing themes, highlighting and discussing areas of overlap and contradiction, (5) refining themes; (6) presenting the work and writing the report brought further clarity to each theme idea.

## Results

Four focus groups were held, involving a total of 30 participants, between February and April 2022 (Table 1).

**Table 1:** Focus group details

Group number	Group description	Online or in-person	Number of participants	Duration in minutes	In-text reference
1	Mothers with lived experience of postnatal mental health difficulties	Online	6	110	FG-1 (Focus group 1)
2	Mothers from the refugee community	In person	12	100	FG-2 (Focus group 2)
3	Mothers with migrant backgrounds	In person	7	75	FG-3 (Focus group 3)
4	Disabled mothers	Online	5	67	FG-4 (Focus group 4)

### Participant characteristics

Ethnicity was reported by 28 of the 30 participants: Black African (n = 8), Arab (n = 5), White British (n = 5), Mixed/multiple ethnic groups (n = 3), Black Caribbean (n = 2), Asian Indian (n = 2), Asian Pakistani (n = 1), Asian Chinese (n = 1), White European (n = 1). Participants were aged between 20 and 42 years, with most mothers in their late 20s and early 30s. Around half of the mothers had more than one child. The age of the youngest infant was 3.5 weeks, with most mothers having a child aged between 4 months and 2 years. There was often overlap between groups; for example, one mother in FG-1 was also from a migrant background and disabled. All participants in FG-1 self-reported some form of postnatal mental health difficulty at the recruitment stage. We also found that high proportions of women across all other groups reported a range of postnatal challenges during the course of the discussions, including depression and anxiety, personality disorder, post-traumatic stress disorder, birth trauma, female genital mutilation, domestic violence, substance use, homelessness, loneliness and isolation, grief, single parenthood and disability (including multiple sclerosis, visual impairment, neurological disability and mobility difficulties).

### Key themes

Analysis of the data resulted in the development of six key themes related mothers’ experiences of being in nature (Table 2). Quotations have been reproduced below to illustrate some of the points made, and tagged to indicate from which focus group they originated.

**Table 2:** Summary of key themes

1.	‘That feels like nature to me’: Mothers’ experiences of what constitutes ‘nature’
2.	‘You can feel that it’s different’: Sensing nature improves wellbeing
3.	‘I could feel all the oxytocin’: Natural spaces facilitate human connection
4.	‘It’s just this lovely escape once you’re there’: Nature provides relief from the stresses of the indoors
5.	‘A different take on things’: Nature allows new perspectives
6.	‘You know it’s good for you, but it’s really hard to get out’: Postnatal mothers face challenges accessing nature

#### ‘That feels like nature to me’: Mothers’ experiences of what constitutes ‘nature’

Mothers defined being in ‘nature’ as being outside, with most particularly valuing accessible local parks, or even simply stepping outside the home:

I do end up in just the local park at the end of my road ‘cause actually that’s all I can manage, but actually that feels alright. [FG-1]

Indeed, most agreed that nature was not a specific destination, but could be found in simple places:

To be honest, wherever there is a patch of grass where you can just sit down and breathe, that feels like nature to me. [FG-3]

A smaller proportion spoke of further away places, such as ‘by the beach side’ [FG-2] or woodland, which allowed them to feel ‘in the middle of the nature’ [FG-2]. Mothers who had not grown up in the UK sometimes drew comparisons between nature in this country and their home country. However, they too agreed that peace could be found in a variety of natural settings, across different countries.

Back home in Africa, we didn’t have parks. We had wide open spaces, but we didn’t have a slide or a swing, we didn’t have any of that. It was like, the beach is ten minutes down the road… It’s different [nature in England], it’s still peaceful, but it’s different. [FG-3]

A sense of ‘peace and quiet’, where ‘you can’t hear the bustling of the city’ [FG-4], was felt by some to be better captured in wilder natural spaces further away from home.

I think I need to go to nature to really feel peaceful. There’s plenty of parks within walking distance from where I am, well ‘walking distance’ being in inverted commas, but it’s not as peaceful, and I feel to be in nature you need to be somewhere at peace. [FG-4]

#### ‘You can feel that it’s different’: Sensing nature improves wellbeing

Mothers emphasised the value of multi-sensory experiences from being in nature for themselves and their babies.

I go to the park quite regular, even on days it’s cold. Sometimes we sit on the grass with a blanket for like 10 minutes or so, just to feel the breeze, feel the grass, feel the mud, just to be in tune with nature as much as possible. [FG-1]

When mothers experienced these sensory connections with nature and noticed their baby’s senses being stimulated, many reported a resulting sense of admiration and awe for nature’s beauty and vastness.

[in English]: I feel it’s amazing when we go to the nature, because it’s beautiful to see, the blossom you can see in the spring… the leaves it’s grow, and in autumn they are fall all away. Autumn colours in nature. Yeah, nature is amazing, yeah. [FG-2]

The diversity of ways in which mothers connected their senses with the outdoors was powerfully captured by disabled mothers. This group commented on how, when one sense is impaired, other senses ‘almost benefited as a result’, and how this can in some ways be ‘more heart-breaking, but in some ways it’s more beautiful’ [FG-4].

P5: I’m visually impaired… when I go out into nature, it’s not so much about the seeing, but the being in nature, it’s about the sounds and the smells and my personal feeling rather than actually how beautiful it looks, for example.

P1: For me I think it’s tactile, being able to feel grass, being able to dip my feet in water or touch a rock or something, or just feel some bark, and I get that quite a lot with [baby’s name] as well. [FG-4]

Although changes in seasons and weather conditions sometimes posed challenges, some mothers described changes in the elements as opportunities to awaken their senses in a different way.

I’ve been for a few walks in the rain there [local cemetery with wooded areas], and it's almost like, magical, being in amongst that forest, while the like, pitter patter of the rain, like all of the senses - and sounds that you can pick up whilst you’re strolling through. [FG-1]

Opportunities for sensory stimulation and fresh air were also credited for improving babies’ sleep, which also enhanced the mothers’ own sleep. Indeed, the wind, the fresh air, and the breeze were cited as important by most mothers across the focus groups. One mother who had experienced severe postnatal anxiety considered access to fresh air as essential to managing the symptoms she faced.

One of the things that I really struggled with was controlling my breath when I was unwell, so if I would speak, I’d lose control of my breath. I found it so much easier to breathe in open spaces, so if I was having a panic attack I would head straight outside. I think it is that sense of, kind of, the fresh air. Again, I don’t know whether it’s a change in temperature or whatever it is, but I found that really useful. [FG-1]

Many of the mothers described finding it relaxing and calming to *feel* the fresh air against their skin. Refugee mothers in particular described how ‘it’s helpful for your body when you have air’ [FG-2], and how ‘it relieves headaches’ [FG-2]. Mothers attributed a sensory connection with nature to imbuing a sense of awe and wonder, which was in turn felt to fuel the imagination and improve wellbeing. The quotations below illustrate how the theme of sensory connection with nature encapsulates both bodily senses and a sense of amazement:

For me personally I love the air, the breeze is beautiful, being able to relax and having this nice fresh air coming at you, it’s amazing, it is, it is. [Agreement from other participants, ‘Oh yeah the breeze!’] [FG-3]

[Translated from Arabic] When you walk in nature, you see the views outside, different trees, and especially in the summertime where we see different flowers. When you contemplate nature, you get a totally different feeling. It’s good for the imagination, good for wellbeing you know. [FG-2]

#### ‘I could feel all the oxytocin’: Natural spaces facilitate human connection

Mothers across all of the focus groups described how nature created a space in which they could connect with other people and with their baby. Meeting people with shared experiences felt particularly important during the postnatal period, in which mothers commonly reported feelings of isolation and loneliness.

For many, the expansive and yet psychologically containing space created by nature also facilitated a different way of communicating with friends and loved ones.

P4: [When we lived in a caravan] we found that just going on a walk in nature we would completely open our minds to a whole different conversation that we weren’t able to even indulge in inside, and I’ve felt the same now with having a baby—you end up just talking about the same things, and then when you go outside and “oh!” there’s a whole new different perspective of things you can talk about…

P2: Yeah, talking and walking always kind of feels easier, I don’t know whether it’s because you don’t have to make eye contact, or if you’re focussing on something else, but it feels like, when you’re walking next to someone, you kind of, you’re able to say different things or communicate differently.

P4: Yeah, you almost feel like there’s a floodgate that’s able to be opened and you’re actually like “yeah I can say this thought-provoking thing and it will be alright”, but when you’re like stuck indoors it’s like “oh no, if I say this, you’re gonna be like stuck in the same room”.

P2: You get trapped in with it, don’t you. [FG-1]

Mothers’ accounts indicated a facilitatory component of nature in fostering closeness with others.

It’s one of the most relaxing spaces when you’re in nature, and I think it’s just an opportunity for people really to get to know each other. [FG-4]

They also conveyed the sense that natural surroundings created a special space for treasured connections to develop with their babies and children, allowing mothers and babies to build their relationship through mutually enjoyable shared experiences. Opportunities for bonding in nature were attributed to a context conducive to joint attention and synchrony, and to feeling happier and more relaxed in each other’s presence when outdoors.

I was there yesterday [in an inner-city park] and they’ve got like a garden area that they grow herbs and stuff… I could feel all the oxytocin going through my body holding [baby], being like “this is sage and this is a tree” [in a cheerful voice] and he’s like babbling away, it was really nice […] Like you know he has no idea of what I’m necessarily talking about when I’m naming random plants and stuff, but he is **there** you know, he’s listening, and it’s nice for him to actually be interested in what I’m saying because there’s other surroundings. [FG-1]

#### ‘It’s just this lovely escape once you’re there’: Nature provides relief from the stresses of the indoors

Although most participants felt that ‘there is something’ [FG-1] intrinsic about nature itself that helps wellbeing—something intangible and ‘difficult to put into words’ [FG-4]—some wondered whether it was the escape from the indoors that was instrumental. Mothers in all groups expressed how oppressive the home environment could come to feel during the postnatal period. They described tasks ‘piling up’ [FG-1, FG-3], babies and children getting ‘agitated’ indoors [FG-1, FG-2], feeling bored, ‘trapped’ [FG-1, FG-3], ‘stuck’ [FG-2] and ‘enclosed in four walls’ [FG-1], feeling exhausted by the constant goal-orientated attention required in the indoor environment.

P3: If I’m home I think ‘I should be cooking, I should be cleaning, I should be getting ready for school’

P6: And I think especially when you live in a flat and you don’t have a garden and you go out in the park and things like that, it’s nice, you have fresh air, ‘cus the flats are small here and you are just sat inside like… uuurgh [motion of head exploding]

P2: And it’s the same four walls. [FG-3]

Mothers from refugee backgrounds used similar language to reflect the oppressive feeling of caring for children indoors, with many describing how getting outside alleviates this stress.

[Translated from Arabic] I like to walk in the open air instead of sitting at home, where the house is small, and you are there where the baby is still small, it’s like it squeezes you inside [you feel pressurised mentally: translator’s note]… I enjoy that time outdoors, you would be relaxed then. [FG-2]

Several also commented on how escaping the indoor environment was important for their children and their relationship with their children. The mother of a four-month-old described important opportunities for distraction when escaping the indoor environment, to the fervent agreement of other participants:

If I’m like struggling indoors, or he’s agitated or I’m agitated, the best medicine is to go outside and just have some fresh air, and walk, and it’s all about distraction as well, distracting him, distracting me. [FG-1]

#### ‘A different take on things’: Nature allows new perspectives

Across all of the focus groups, mothers conceptualised natural environments as highly conducive to their wellbeing in the postnatal period, allowing sometimes profound shifts in perspective: altering their views of themselves, their thoughts and feelings, their priorities, and even their take on life.

Mothers described how spending time in nature with their baby encouraged happiness and joy, interest and fascination, relaxation, calm, peace, feeling ‘grounded’ [FG-1], feeling soothed, as well as reducing stress and lifting depressed moods. These positive psychological states then allowed a feeling of restoration, as if spending time in nature is ‘like pressing reset’ [FG-1], like ‘therapy’ [FG-4], or ‘like the energy’s coming back’ [FG-2].

One mother poignantly described how critical such changes of perspective could be when experiencing severe postnatal mental health difficulties, and how these shifts facilitated emotional self-regulation.

Especially if you’re unwell - actually it’s really hard to leave the house - and so if you do manage to kind of get out of the house, then actually it does make you feel better, and I feel that you get that sense of perspective a little bit more, it’s easier to kind of [pause…] talk yourself down from a ledge if you’re outdoors. [FG-1]

Mothers with lived experience of postnatal mental health difficulties described achieving an enhanced sense of self-efficacy, confidence, achievement, and empowerment through spending time in nature with their baby. This was particularly acute when they had faced a challenge, such as going out despite adverse weather conditions.

P6: Today I did go out in the rain, both of us got absolutely soaked, my socks were like sodden half way up my feet, but I was like, “I did though, I still did it! [triumphantly] and it was fine”.

P3: It’s way more empowering when it’s not your ideal situation and you’ve actually done it. [FG-1]

Shifts in visual and auditory perspective were described as important in the context of the demands of looking after a baby, and link to the previous idea of how a change in physical perspective can feel freeing, soothing, and restorative.

[in English] And your eyes… it’s nice to see a lot [motions far away into the distance]…it’s like, green, light, lots of things. [FG-2]

There’s something about being outdoors. My firstborn was really refluxy and he used to cry all the time, and that being stuck in the house just hearing him cry, well it sends you to like different levels of crazy doesn’t it [agreement from others], you’re like “I can’t do this, I can’t do this”. And then you get out of the house and suddenly they don’t sound as loud, it sounds really bad, but it, like, tones them down a notch, and it’s like “okay, alright, we can do this, it’s okay”. [FG-1]

One mother described how her perspective on time changed when out in nature, which alleviated the boredom of the day and allowed her to feel restored.

When you’re sat in the house, I’m constantly like… how can I make this day shorter? It’s like this day is going on forever, like what can I do? “Let’s get out in nature!”, you go outside and an hour and a half’s gone by, you know time just goes a lot quicker because you’re distracted by everything around you… you’ve both got quality fresh air and come back, you reset. [FG-1]

#### ‘You know it’s good for you, but it’s really hard to get out’: Postnatal mothers face challenges accessing nature

Mothers across the different focus groups, from a range of backgrounds, seemed aligned on the wellbeing benefits of nature. Where each group diverged more in its discussions was when considering barriers to accessing nature. These barriers were often specific to the postnatal period. For example, women differed in their views about whether breastfeeding facilitated or hindered getting outdoors, with some feeling ‘self-conscious breastfeeding in public’ [FG-1], and others feeling like ‘a free mum’ [FG-3].

All groups voiced concerns about adverse weather conditions when caring for small babies, such as cold, wind and rain. A minority disagreed; for example, in FG-1 and FG-2, some felt that rain could enhance the repertoire of sensory experiences in nature. Many mothers described not wanting to go out in the rain, fearing getting wet whilst waiting for public transport, the baby ‘catching a cold’ [all groups], and the difficulties around wrapping babies in waterproofs. Mothers who had grown up in hot countries especially considered the cold a barrier to being in nature:

We don’t like cold. I’m coming from a very hot country. Here, you feel it’s always winter. In Britain, it is always winter. [FG-2]

Other practicalities, such as transport and facilities, took on new significance when factoring in a new baby. Many living in the inner city did not drive, and felt that public transport was too costly, unreliable, or difficult to be able to access nature with their baby. Although accessibility issues affected most mothers in the postnatal period, the theme came through particularly strongly in the group for disabled mothers.

That’s another thing that you have to overcome before you even start going into nature and stuff, and I just think there’s a lot as a disabled person that you have to face that you have to think about, like toilets, or accessibility to different things. [FG-4]

One disabled mother described the impossibility of getting onto a bus with a pushchair, reflecting the added logistical complexity experienced by many disabled mothers on account of trying to manage their impairments as well as a new baby. Most participants emphasised feeling overwhelmed by the practical and mental preparation required to get out of the house in the first place with a baby, and particularly those struggling with mental health difficulties.

There’s the fear of the unknown as well, so if you don’t trust your own body and you don’t trust your own brain to give you the right cues, what if something scares you when you’re out, or what if something triggers your anxiety whilst you’re out, what will happen then? And like, “will I be safe?” and “how do I look after myself and my baby?”. So I think there’s quite a practical element to the logistics, but then there is also that, kind of, fear of not trusting yourself and your own, sort of, mind I suppose. [FG-1]

Psychological barriers to leaving the house were compounded by, and linked to, physical problems in the postnatal period. These included birth-related complications, such as having had a caesarean section or episiotomy, or complications related to prior female genital mutilation in certain cultural groups, restricting physical mobility.

Many refugee mothers faced cultural considerations relating to leaving the house in the early postnatal period. The traditions of many included the practice of forty days of rest at home following childbirth, during which time mothers would recuperate from birth, bond with the baby, and receive help from elder female family members. However, many mothers described the need to adapt to the host country, in the absence of extended support, which resulted in leaving the house much sooner. Many migrant mothers also lamented the absence of extended family support, which they related to feeling alone and unsafe in their neighbourhood.

P3: We’re African, we are outside a lot back home, we’re not indoors […] It’s different here, you’re thinking, is your child going in a gang, or is someone looking at your child to groom them. But in Africa like I said, it’s different.

P1: I think in Africa it is very cemented that it takes a village to raise a child, whereas here you are by yourself. [Strong agreement from the whole group]

P5: It’s just you and your children here. [FG-3]

Mothers from diverse ethnic backgrounds experienced racist comments in certain parts of the city, making them reluctant to explore unfamiliar places, including natural environments. Refugee mothers also experienced stress when other families were not welcoming:

P6 [Translated from Arabic]: It’s nice to be outside, but sometimes I’m worried because when my kids go to play with other families, maybe the parents are not happy or welcoming that, and that just makes me more worried in the park.

P2 [In English]: That’s true. This happens a lot. There are families you feel that they put up walls, the reaction they have us not happy, so you feel that you must keep your child tied to you. [FG-2]

## Discussion

To our knowledge, this study is the first of its kind exploring the effects of spending time in nature during the postnatal period, from the perspectives of mothers from diverse backgrounds. The main findings were as follows: (1) What constitutes ‘nature’ depends on mothers’ circumstances, but most value access to peaceful, nearby nature during the postnatal period; (2) Mothers experienced wellbeing benefits from connecting with nature using the senses, which in turn led to a deep appreciation for nature’s beauty and wonder; (3) Nature created a favourable space for mothers to form human connections, both with their baby and other people; (4) Nature provided an escape from the stressful indoor environment and daily tasks; (5) Time in nature allowed powerful shifts in perspective which were restorative and conducive to wellbeing; (6) Mothers faced specific environmental, practical, psychological, socioeconomic and cultural barriers to accessing nature during the postnatal period. These findings may be useful for determining how to support postnatal mothers, and are therefore discussed in these terms below.

Mothers’ appreciation for nearby, accessible, peaceful, safe natural spaces reflects wider research highlighting the importance of proximity to green space [50, 51]. Research has found a positive association between pregnant women living near green spaces and improved birth outcomes including birthweight [52, 53]. During the COVID-19 pandemic, residential proximity to green spaces was found to be associated with a buffering effect on stress responses in mothers of young children [41]. Furthermore, mothers’ perceived proximity to green space is even associated with TV viewing time in children, with higher weekly TV time in children living furthest from green spaces [50]. Mothers’ descriptions of ‘nature’ in our study ranged from wide open green spaces and woodland, to a simple patch of grass, and to occasional visits to so-called ‘blue spaces’ such as the seaside. We also found that, although some mothers in this study had grown up abroad in their home countries, they were still able to find peace in the natural spaces of the UK.

The sensory connection experienced in natural settings was perceived as highly conducive to wellbeing. Indeed, sensory contact with nature has been conceptualised as one of the ‘Five Pathways’ to nature connection [54]. These five pathways also include Beauty, Meaning, Connection, and Compassion, several of which also featured within our participants’ accounts of time spent in nature in the postnatal period. Engagement with natural beauty, for example, has been found to mediate the relationship between nature connectedness and happiness [55]. A visual appreciation for nature was reflected by mothers describing the relaxing properties of the colour green, which in other research has been described as a low-arousal, low-anxiety, and highly preferred colour [56]. This is in contrast to the grey colours of urban scenes which trigger more negative emotions [56]. Research with disabled participants, seeking to challenge ableist assumptions that often underpin popular discourses of nature, also encourage appreciation of ‘richly textured ways’ in which people experience nature, beyond the visual [57]. Mothers in our study, including disabled mothers, emphasised the importance of sounds, smell, and touch experienced in nature. In keeping with our participants’ accounts, a preference for sounds of nature (such as wind, water, and animals) has been repeatedly found over anthropogenic sounds such as traffic and industrial noise [58]. Relevant to our population of interest, listening to nature-based sounds has been found to reduce pain after caesarean section [59].

Previous research suggests that it is not the amount of time spent in nature, but rather noticing and engaging with nature through simple activities that is most beneficial [60]. Examples include listening to bird song or smelling wildflowers [61], as described by the participants in our study. Natural smells have been found to underpin profound experiences of nature for many people, a phenomenon which may have roots in evolutionary psychology. Smells (including the notoriously evocative smells of freshly-cut grass or damp earth) can have important effects on our mood, behaviour, and cognition [58]. Use of herbal therapy, for example smelling lavender oil aroma, has a rich tradition of use among postpartum women from diverse cultures, and a tentatively emerging evidence base [62]. Connecting with nature through touch is an under-researched area, but mothers in our study described the positive effects for example of touching a rock, dipping one’s feet into water, or being in physical contact with grass, mud, and the breeze.

Our findings support the notion that natural environments, which offer space, sensory stimulation, interest, and opportunities for creativity, have the potential to be highly conducive to shared enjoyment between parents and even very young infants. Research has found that environments allowing the infant to be an active partner within the dyadic interaction, for example engaged in mutual enjoyment, fosters positive parent-infant interactions [63]. Research in older children has found associations between time spent in nature and higher positive affect, increased energy, and less anger [64, 65]. Parent–child communication (with 3- to 4-year-olds) has also been found to be more responsive and connected in natural compared to indoor environments [66]. The capacity for shared pleasure, mutual positive affect and communication are important components of the mother-infant dyadic relationship [63], and may be supported by natural environments from an early age. Moreover, given high rates of loneliness and social isolation amongst new mothers, especially from minority groups, the opportunities for interaction with the wider community may be a particularly important component of time spent in nature.

The fourth theme specifies how nature’s previously documented capacity to provide an escape from daily stresses is pertinent to mothers in the postnatal period; the sense of freedom and happiness that being outdoors brings to mothers seems to have profound effects on their wellbeing. ‘Being away’, a reduced sense of urgency, and engaging in a more reflective mode of thought is described as part of Kaplan’s Attention Restoration Theory [67]. This theory posits that concentration and motivation are restored through the impact of nature on attentional processes. The so-called ‘soft fascination’ experienced in nature appears to represent an important break from the goal-directed attention required of parenting, a break which was perceived by our participants as highly restorative. This is in keeping with previous research finding that sustained attention and creative problem-solving are improved after periods of immersion in nature [28, 29, 68]. Even after short trips outdoors, participants in this study reported an increased ability to face the challenges of caring for their infant and household tasks. Mothers’ accounts are also in keeping with self-regulatory theories of nature engagement [20], with time in nature acting to regulate emotions, helping to cope with and buffering the effects of parenting-related stress. The calming qualities of the aesthetic aspects of nature are one of the affective reactions described in Ulrich’s Stress Reduction Theory [69]. More recently, Richardson’s emotion regulation theory (2019) describes neurophysiological pathways for this effect, via balancing of the parasympathetic and sympathetic nervous systems [19].

There were many similarities between all four focus group discussions, especially regarding themes 1 to 5. Facilitators were struck by how all of the women, from very diverse backgrounds, used surprisingly similar terms to describe the wellbeing benefits of being in nature during the postnatal period. Differences between groups were more pronounced around the theme of barriers to accessing nature. Although mothers clearly valued time spent in nature with their infant, a variety of environmental, practical, psychological, physical, socioeconomic, and cultural barriers rendered this challenging. Research has found that the majority of the population spends large amounts of time indoors, including mothers of young children [70]. People from lower socioeconomic groups are less likely to have access to greenspaces with good amenities [71], and our findings also support concerns expressed by mothers about general safety concerns [72] and long walking distances [50], and the importance of neighbourhood safety and social coherence [51]. These factors all influence the likelihood of visits to nature. Participants in the disabled mothers’ group described specific issues related to mobility and poor vision that sometimes affected their access to nature. Participants in the refugee mothers’ group discussed cultural practices that were specific to their countries of origin, which could affect their access to nature, particularly in the early postnatal period. Although all groups agreed that poor weather could be a potential barrier, this was felt more strongly by participants from ethnic minority groups, who were also more likely to express concerns about their babies becoming unwell if taken outside in the cold or rain. Moreover, these participants described experiences of feeling unwelcome by other people in natural environments, potentially affecting their motivation to access nature. Future interventions to promote contact with the natural world in the postnatal period must take these barriers to access into account, including issues around safety and accessibility. The differences between focus group discussion content in our study reinforced the need to seek diversity amongst patient views when considering a healthcare intervention to improve access to nature.

### Strengths and limitations

The use of focus groups with women with shared characteristics generated rich, lively, and free-flowing discussion. Our recruitment strategy successfully identified mothers from diverse backgrounds, and with differing relationships to the natural world, although our sample was primarily urban. The number of participants in each group ranged from 5 to 12. A high number of participants in the group of 12 refugee women meant that each individual had less time to speak, although this was the second-longest focus group. Nevertheless, conversation between participants appeared to flow freely and comfortably. This may have been because the women knew each other already, and because they identified as coming from ‘collectivist’ cultures, in which being part of strong, cohesive groups is important. For all focus groups, flexibility in the discussions was required to accommodate the presence of babies. This allowed participants to respond to their babies and re-join the conversation when possible, although none of the participants needed to leave any of the groups. Whether the focus groups were held in person or online, mothers reported feeling at ease, knowing that other participants were in a similar situation to them. The online forum did not seem to compromise the flow of conversation. Some mothers with mental health difficulties or disabilities reported that groups being held online made them more accessible, both for logistical reasons and because they felt less apprehensive joining remotely. By contrast, some other mothers (for example from the refugee community) mentioned that they were pleased to engage in person, for reasons of preference, not having the digital facilities to access an online group, and to make it easier for the interpreter.

Most mothers across groups identified some sort from ‘postnatal distress’ throughout the discussions, a term reflecting the range of interrelated emotional difficulties experienced at this complex time of life [73]. Given the problem of under-diagnosis of postnatal mental health difficulties, we consider it a strength that we did not stipulate the need for a mental health-related diagnosis for inclusion in this study.

During focus groups, mothers were asked to think about the postnatal period specifically (defined as the first two years following childbirth). Most participating mothers were currently in the postnatal period, but we chose not to exclude those with slightly older children who wanted to take part. Some may wonder whether the accounts from these women may have been more open to retrospective reconstruction, but these mothers gave clear accounts of their postnatal experiences, and indeed the passage of time had given them an opportunity to reflect on them. Nesting our data collection within a nature-based intervention development study may have attracted participants who felt particularly affiliated with nature, given that our sample was self-selecting. This issue was mitigated in the focus groups for refugee and migrant mothers, given that all those who usually attended their weekly groups were happy to take part in the study.

A further strength of this study was the involvement of a highly multi-disciplinary team in the research design, execution, and analysis. The team comprises members with lived experience of postnatal mental health difficulties (RH), experience working with marginalised groups (RM), experience of disability and disability advocacy (RN), nature-based practice (LD), academic expertise on the relationship between nature and health (CB), environmental humanities (SW), perinatal academic expertise (JE), clinical experience (JE) and charity provision (MW).

## Conclusions and future directions

Being in nature can benefit women during the postnatal period, mentally, physically, and socially. It can also help them to connect with their infant. Our findings have implications for supporting postnatal mothers, potentially both in the prevention and treatment of postnatal stresses. One example is by developing tailored nature-based interventions that optimise the benefits described in this study, are cost-effective, sustainable, and transferrable across different contexts, addressing the needs of mothers from different socio-economic and cultural backgrounds and with different personal circumstances. Qualitative findings may inform choice of intervention sites, programme content, and how to maximise inclusivity of minority groups and cross-cultural validity. This study may also inform future research into the impact of nature on women throughout the perinatal period, defined as the time during pregnancy and up to either one or two years following birth.

## Data Availability

The datasets generated during the current study are not publicly available due privacy and confidentiality reasons, but are available from the corresponding author upon reasonable request.
